# Gleet

**Published:** 1860-11

**Authors:** 


### Gleet.

Every one connected with the medical profession, knows something of the difficulty met with in the cure of this troublesome disease, and many sufferers deplore the imperfection of the art, when laboring under this tormenting difficulty.—The "Cincinnati Lancet & Observer" gives the following plan of treatment, as derived from a correspondent of a London journal:

"Take a moderate sized wax bougie (the common yellow wax ones are the best), warm it slightly, and then roll it for a few seconds in well powdered alum; when thoroughly whitened with the salt, roll it between the hands, so as to press the alum well into the wax, and the instrument is ready for use. Make the patient micturate previously, and then pass your bougie, without the assistance of oil or cerate, so far as may be deemed advisable, cutting it off to within an inch of the orifice of the meatus, where it may be tied or not, and left for one hour each day. In this way a tiresome and refractory old gleet may be cured in ten days."

